# A tree search algorithm towards solving Ising formulated combinatorial optimization problems

**DOI:** 10.1038/s41598-022-19102-x

**Published:** 2022-08-30

**Authors:** Yunuo Cen, Debasis Das, Xuanyao Fong

**Affiliations:** Department of Electrical and Computer Engineering, National University of Singapore, Singapore, 117583 Singapore

**Keywords:** Computer science, Information technology

## Abstract

Simulated annealing (SA) attracts more attention among classical heuristic algorithms because many combinatorial optimization problems can be easily recast as the ground state search problem of the Ising Hamiltonian. However, for practical implementation, the annealing process cannot be arbitrarily slow and hence, it may deviate from the expected stationary Boltzmann distribution and become trapped in a local energy minimum. To overcome this problem, this paper proposes a heuristic search algorithm by expanding search space from a Markov chain to a recursive depth limited tree based on SA, where the parent and child nodes represent the current and future spin states. At each iteration, the algorithm selects the best near-optimal solution within the feasible search space by exploring along the tree in the sense of “look ahead”. Furthermore, motivated by the coherent Ising machine (CIM), the discrete representation of spin states is relaxed to a continuous representation with a regularization term, which enables the use of the reduced dynamics of the oscillators to explore the surrounding neighborhood of the selected tree nodes. We tested our algorithm on a representative NP-hard problem (MAX-CUT) to illustrate the effectiveness of the proposed algorithm compared to semi-definite programming (SDP), SA, and simulated CIM. Our results show that with the primal heuristics SA and CIM, our high-level tree search strategy is able to provide solutions within fewer epochs for Ising formulated combinatorial optimization problems.

## Introduction

Many combinatorial optimization problems (*e.g.*, VLSI floorplanning^1^, drug discovery^2^, and advertisement allocation^3^) aim to find the optimal solution among a finite set of feasible solutions. However, finding the exact solution to combinatorial optimization problems generally requires the exploration of the entire solution space, which increases exponentially in size with the size of the optimization problem and makes it intractable to solve exactly^4^. As a result, the research community has a significant interest in algorithms to find near-optimal solutions within a reasonable time.

**Figure 1:**
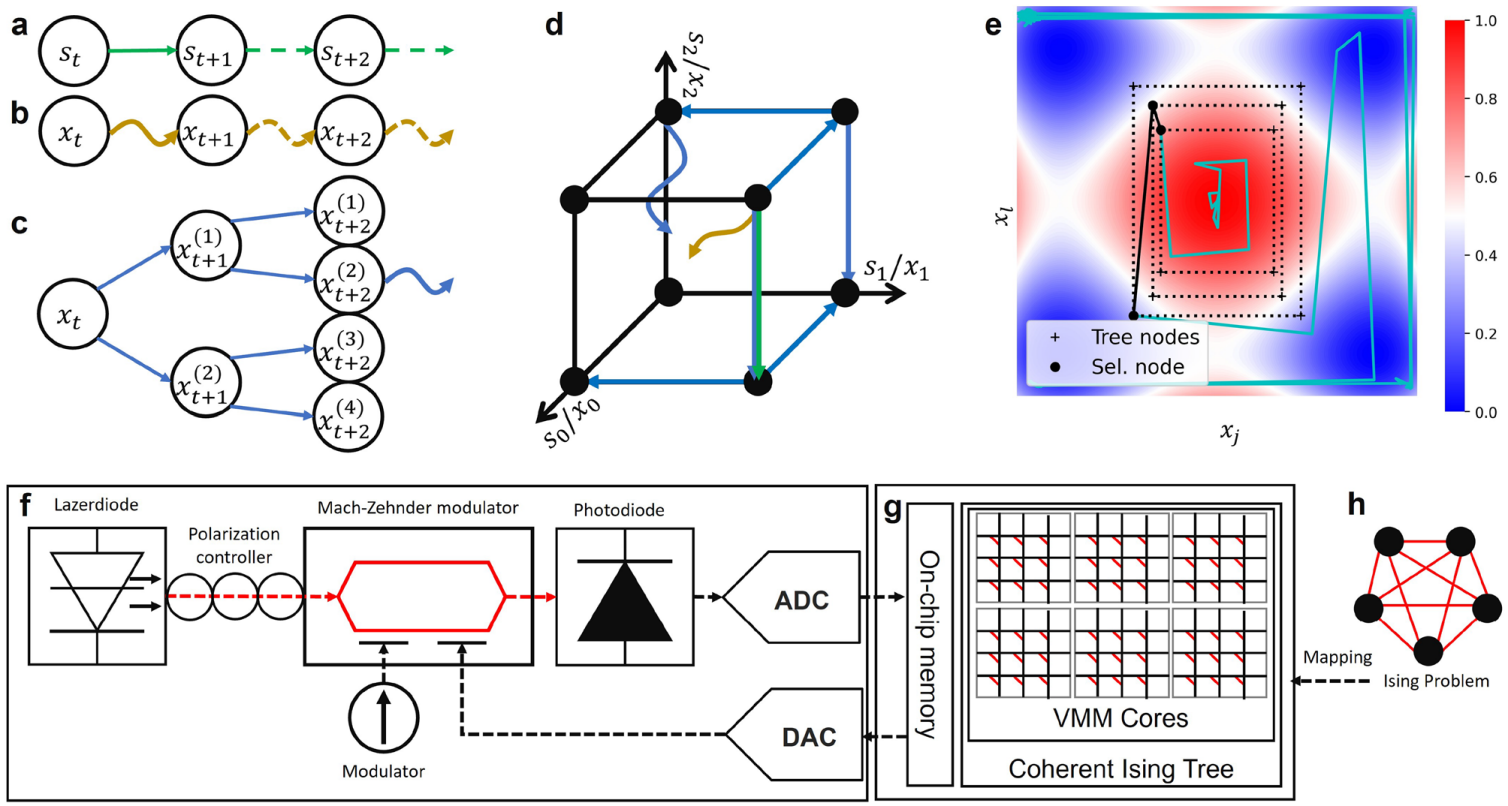
Search space of SA, CIM and CITS within coupled Ising spins: **(a)** The Markov chain of SA, where the straight solid/dot lines represent the Metropolis-Hasting sampling at current/future time step. **(b)** The Markov chain of CIM, where the curve solid/dot lines represent the oscillator dynamics at current/future time step. **(c)**. The Ising tree structure of CITS, where the straight lines represent the expansion based on SA and the curve line represents the exploration based on CIM; **(d)** Intuitive explanation of annealing mechanism of CITS. From the initial spin configuration with high Ising Hamiltonian, expanding the search space by primal heuristic SA (blue straight lines), then exploring the two local spaces in parallel by primal heuristic CIM (blue curve lines). **(e)** Explanatory annealing process on two uncoupled Ising spins. Each dashed triangle represents the child nodes of the corresponding time step. Only the most potential node is selected for future time steps. **(f)** Experimental schematic of poor man’s CIM^21^, where we abstract and simulate the reduced dynamics since we only care about the solution quality instead of measuring the whole system. **(g)** Hardware design of CITS interfaces with poor man’s CIM. The VMM cores compute the Hamiltonian and the reward of the child nodes. This part can be paralleled and accelerated using FPGA or non-volatile memory technology. The on-chip memory stores the Ising spin configurations of each node, and the corresponding Hamiltonian. **(h)** Ising formulation of combinatorial optimization problems that can map the edge values to the cells in VMM cores.

Among the approaches in the literature, the Ising model (Fig. 1h) has attracted the most attention because it is straightforward to recast many combinatorial optimization problems as the problem of searching for the ground state of an Ising Hamiltonian^4^. Moreover, two well-known heuristic algorithms have been studied extensively: quantum annealing (QA)^5^ and its classical counterpart, SA^6^. In these algorithms, the cost function is directly encoded as the Ising Hamiltonian and feasible solutions to the combinatorial optimization problems are obtained by searching for the energy minima. Furthermore, it can be theoretically shown that QA and SA can obtain the exact solution if the annealing time is large enough^7^. However, the QA is usually implemented on superconducting qubits, which can be costly. On the other hand, the Ising spins for SA can be hardware-friendly for conventional computers^8^, especially for CMOS-compatible spintronics implementation^9–12^. Regardless of the implementation, achieving the Boltzmann distribution using the annealing process may become intractable. It may be more practical to achieve a quasi-equilibrium distribution by considering only the flipping probabilities of identical spins. However, such an approach usually traps the SA in a local optimum^13^. Some studies try to avoid this situation by introducing noise^11^ but the effectiveness of noise injection is still under debate.

The CIM has garnered much research interest because its bistable coherent states can be naturally mapped to the Ising Hamiltonian^14^, and is efficient for sampling spin configurations (which also are the solutions to the combinatorial optimization problems)^15^. Compared to SA, CIM has the theoretical advantage of facilitating a quantum parallel search across multiple regions^16^. Compared to QA, CIM has the physical advantages of room temperature operation, scalability, and connectivity^17^, which arises from the fact that the information loss due to the measurement-feedback scheme for capturing the reduced dynamics of CIM seems to be unimportant^18^. Based on this scheme, the research community has scaled up the size of CIM from the initial 4 spins^19^ to 100,000 spins^20^. However, the cost of scaling up the CIM is not cheap, and the effort to stabilize a large CIM may not be trivial. The poor man’s CIM (as depicted in Fig. 1f) offers significant advantages in stability, size and cost, and can achieve similar performance in terms of success rate^21^. However, in the measurement-feedback scheme, measurements need to be made from the optical systems and converted to a digital signal for the field-programmable gate array (FPGA). After the calculations in FPGA are completed, the results are converted back to an analog signal and injected back into the optical system. The data conversion and movement between analog and digital domains is a severe bottleneck for the computational performance and hence, it is desirable to reduce the number of these processes. Doing so reduces the epochs-to-solution and achieves speedup for the CIM to find a near-optimal solution. Table 1 summarizes the main contribution of this work with respect to the related works in the literature.

**Table 1:** Remarks of related works and main contribution of this work.

CIMs	Remarks
DOPO^14^	The reduced dynamics of the degenerate optical parametric oscillators (DOPO) from the Heisenberg-Langevin equation is derived
CIM^19^	Coupling of 4 degenerate optical parametric oscillators with network of delay lines is achieved
MFB-CIM^18^	The coupled degenerate optical parametric oscillators based on measure-feedback (MFB) scheme is scaled up by replacing the networks of delay lines with FPGA
MFB-CIM^20^	The scalability of the measure-feedback based degenerate optical parametric oscillators is demonstrated up to 100,000 nodes
Poor man's CIM^21^	Size, cost and gain stability with optical opto-electronic oscillator and measurement-feedback scheme are improved
This work	Based on opto-electronics oscillators and measurement feedback scheme, a tree search algorithm to gain speed up in terms of epoch-to-solution is designed

To overcome the aforementioned challenges, we propose a tree search algorithm, which we call as coherent Ising tree search (CITS), that combines two primal heuristics (SA and CIM) to find near-optimal solutions. The proposed algorithm is inspired by the Monte Carlo tree search (MCTS), which is a best-first search algorithm that expands the search tree based on random exploration and takes the most promising move^22^. CITS is a recursive size limited search algorithm following the idea of “look-ahead”. The main differences between CITS and MCTS are as follows:

- MCTS expands child nodes for all possible moves at each time step, and the overall tree is needed for the future time steps. The expansion step of CITS is as follows: the child node corresponding to the selected spin state becomes a root node, and the unselected nodes are pruned away, eventually expanding the child nodes to a tree (limited to predefined depth and breadth) based on the primal SA heuristic. At each time step, CITS generates a size limited tree and hence, it is a recursive size limited search algorithm;
- MCTS only visits one path in the tree from the root to the leaf node at each time step while CITS searches the entire Ising tree;
- MCTS is usually applied to zero-sum and complete information games so each leaf node has an exact reward value. CITS is applied on combinatorial optimization problems in which the best optimum is not known. Thus, the change in Ising Hamiltonian determines the leaf value;
- MCTS predicts the leaf value by performing a playout with random moves to end the game whereas CITS evaluates the Hamiltonian of the Ising spin configurations of all child nodes and the rewards of child nodes are backpropagated to their parent nodes;

In CITS, a Markov chain in conventional SA or CIM is expanded to an “Ising tree”, where the future spin states are also taken into consideration (Fig. 1a–c). Instead of simply searching for a lower Ising Hamiltonian in Stone space $\left( \{-1,+1\}^n\right)$ using SA or in real coordinate space $\left( \mathbb {R}^n\right)$ based on the reduced dynamics of CIM, the high-level strategy of CITS leverages both primal SA and CIM heuristics in both search spaces to obtain solutions of the Ising formulated problems (Fig. 1d). The dashed rectangles in Fig. 1e describes the depth and the breadth of our CITS algorithms. At each time step, only the most prominent node is selected for the future time step. Particularly, the computation speed when utilizing the measurement-feedback scheme is mainly limited by the communication time between analog (Fig. 1f) and digital domain (Fig. 1g). As a result, linearly scaling up (tree depth *d* and breadth *b*) the computation in the digital domain will require more computational resources, especially the amount of vector-matrix-multiplication (VMM) cores and the size of memory. However, the total time of each cycle will not increase too much as long as the memory can be realized on-chip, such that the computation time, *i.e.* on FPGA, can be orders of magnitude smaller than the communication time. In this paper, we demonstrate that CITS tradeoffs an acceptable spatial complexity to accelerate the search to find better solutions to MAX-CUT problems (as compared to SA or CIM) with graph sizes ranging from 36 nodes to 230 nodes.

The rest of the paper is organized as follows. In the “Results” section, we first discuss the reduced dynamics of the coherent Ising tree search in terms of the Lagrange picture and relate CITS to the poor man’s CIM^21^. We also study two search schemes to show that the key parameters that determine the performance of CITS are the breadth and the depth of the tree. Results of the evaluation of our proposed methods on different MAX-CUT instances to benchmark with the primal heuristics are also presented. In the “Discussion” section, we summarize our proposed method and discuss the threat to the validity (since we did not formulate the exact noise in our simulations). Discussion about the future work beyond CITS is also presented. Finally, further details of our proposed methods are provided in the “Method” section.

## Results

In this section, we first attempt to visualize the reduced dynamics in the Lagrange picture so as to show how CITS is able to explore and exploit the search space. However, since the exhaustive exploration of every search space is not feasible, we proposed a naive search scheme, which is presented in a later subsection. Results of the ablation study show that the original scheme and naive search scheme generate solutions that are similar in quality. The preceding subsection shows the benchmark results on different MAX-CUT instances with sizes ranging from 36 to 230. And our algorithm details can be found in the “Methods” section.

### Reduced dynamics of coherent Ising tree in the Lagrange picture

The Ising model (Fig. 1c)) describes the behaviour of Ising spins and interactions between each other. The general form of the Ising Hamiltonian may be written as^23^:

1 $$\begin{aligned} H\left( s_1,s_2,...,s_n\right) =-\frac{1}{2}\sum _{j}^{n}\sum _{l}^{n}J_{jl}s_{j}s_{l} \end{aligned}$$

$s_j$ is the state of the $j^{th}$ spin in the Ising model, which only has two states (spin-up or spin-down) represented by $+1$ or $-1$. $J_{jl} \left( =J_{lj}\right)$ is the coupling coefficient between the $j^{th}$ and the $l^{th}$ spins, and represents ferromagnetic (antiferromagnetic) coupling if it is positive (negative)^24^. For any Ising formulated combinatorial optimization problem, the aim is to encode the problem with an Ising Hamiltonian and search for a spin configuration that minimizes the Hamiltonian^4^. Alternatively, the Ising spins can also be modeled as wave functions. The phase differences may then be used to represent spin-up (0-phase) or spin-down ($\pi$-phase). Note that additional constraints are required for the phase degeneracy of Ising spins when mapping from combinatorial optimization problems, *i.e.* second harmonic injection locking^25^ for classical approach or down conversion^14^ for quantum approach. In our approach, we start from quantum harmonic oscillators, $\hbar \omega _0\hat{a}^\dagger \hat{a}$, and a parametric nonlinear (trigonometric) feedback signal from the external field is utilized to degenerate and couple the Ising spins:

2 $$\begin{aligned} \hat{H}\left( \hat{a}_1,\hat{a}_2,...,\hat{a}_n\right) = \sum _j^n\left[ \hbar \omega _0\hat{a}_j^\dagger \hat{a}_j+ \hbar \omega _\alpha \cos \left( \frac{\omega _0}{2\omega _\alpha }\left( \hat{a}_j^\dagger +\hat{a}_j\right) + \frac{\omega _0}{2\omega _\beta }\sum _l^nJ_{jl}\left( \hat{a}_l^\dagger +\hat{a}_l\right) \right) +\Xi \right] \end{aligned}$$

where $\left( \hat{a}^\dagger , \hat{a}\right)$ are the creation and annihilation operator, $\hbar$ is the Dirac constant, $\omega _0$ is the intrinsic frequency of the Ising spins. $\omega _\alpha$ denotes the frequency of the injected feedback signals that pump the Ising spins into two coherent states, $\omega _\beta$ denotes the frequency of the mutual coupling signals encoding the Ising Hamiltonian as shown in Eq. (1). $\Xi$ is the random diffusion term modelled as 0-mean Gaussian white noise.

The quantum-inspired algorithms excavate the potential for solving combinatorial optimization problems by leveraging the underlying reduced dynamics of Hamiltonian systems. Based on the Heisenberg equation, the motion of the annihilation operators can potentially describe an optimization pathway toward the global optimum. Utilizing the Langevin equation, the Hamiltonian in Eq. (2) can be translated into the Heisenberg picture^14^. A classical approach is to approximate the expectation of the Hamiltonian by $\langle \Phi |\hat{H}|\Phi \rangle$ using the complex representation $\Phi =[\phi _j]=[x_j+iy_j]$^26^. Then, the Lagrangian captures the classical approximation of the reduced dynamics of two quadrature components. As a result, the reduced dynamics of the Ising spins in the Lagrange picture becomes:

3 $$\begin{aligned} \begin{aligned} \frac{\partial x_j}{\partial t}&=-\lambda _x\frac{\partial \langle \Phi |\hat{H}|\Phi \rangle }{\partial x_j}\ =\hbar \omega _0\sin \left( 2\alpha x_j+2\beta \sum _{l=1}^nJ_{jl}x_l\right) -2\hbar \omega _0x_j+2\hbar \omega _0\frac{d\xi _{x,j}}{dt}\\ \frac{\partial y_j}{\partial t}&=-\lambda _y\frac{\partial \langle \Phi |\hat{H}|\Phi \rangle }{\partial y_j} =-2\hbar \omega _0y_j+2\hbar \omega _0\frac{d\xi _{y,j}}{dt} \end{aligned} \end{aligned}$$

where $\lambda _x, \lambda _y(=1)$ are the Lagrange multiplier of the *x* and *y* components. $\alpha =(\omega _0/2\omega _\alpha )$ is the feedback gain and $\beta =(\omega _0/2\omega _\beta )$ is the coupling gain. After discretizing Eq. (3) using the Euler method with $\Delta {t}=1/2\hbar \omega _0$, the reduced dynamics are mathematically equivalent to the poor man’s CIM^21^ if the Gaussian white noise is neglected. Note that, in the rest of this paper, we use a discrete version to model the reduced dynamics in sense of the measurement-feedback approach.

4 $$\begin{aligned} \begin{aligned} x_j[t+1]&= x_j[t] + \frac{\partial x_j[t]}{\partial t}\Delta {t} =\frac{1}{2}\sin \left( 2\alpha x_j[t]+2\beta \sum _{l=1}^nJ_{jl}x_l[t]\right) +\xi _{x,j}[t] \\ y_j[t+1]&= y_j[t] + \frac{\partial y_j[t]}{\partial t}\Delta {t} = \xi _{y,j}[t] \end{aligned} \end{aligned}$$

**Figure 2:**
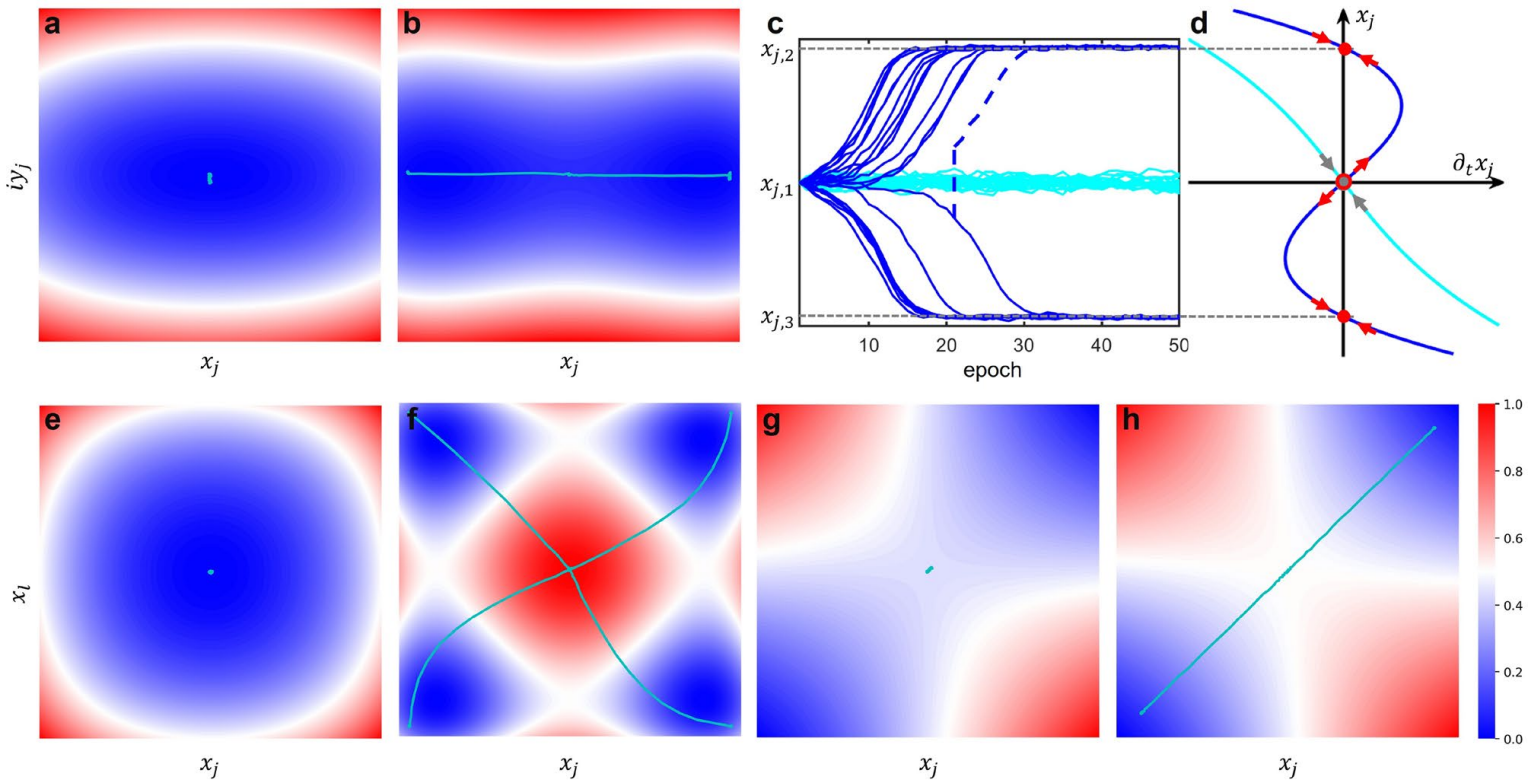
Reduced dynamics of parametric nonlinear (trigonometric) oscillator: **(a, b)** Energy landscape and trajectory of uncoupled Ising spins when increasing the feedback gain from below the threshold ($\alpha =0.8$) to above the threshold ($\alpha =1.3$). **(c)** Time evolution of the real part of the reduced dynamics with feedback gain $\alpha =0.8$ (cyan) and $\alpha =1.3$ (blue), where the dash line indicates flipping the sign of the spins will not affect the bistability. **(d)** Stability analysis of the uncoupled spins. When the feedback gain is below the threshold, the real part of the reduced dynamics will only have one stable fixed point at $x_1 = 0$ (gray dot and arrow). When the feedback gain is above the threshold, the stable fixed points at $x_1 = 0$ become unstable (red ring and arrow), and there exist two symmetric stable fixed points at $x_2 = -x_3$ (red dot and arrow). **(e, f)** Projected energy landscape on real axis and trajectory of two uncoupled Ising spins when increasing the feedback gain from below the threshold ($\alpha =0.8$) to above the threshold ($\alpha =1.3$). **(g, h)** Projected energy landscape on real axis and trajectory of two coupled Ising spins when increasing the feedback gain from below the threshold ($\alpha =\beta =0.5$) to above the threshold ($\alpha =\beta =0.6$).

$x_j[t],y_j[t]$ are the pair of canonical conjugate variables for the $j^{th}$ Ising spin with time derivatives of $\partial x_j[t]/\partial t,\partial y_j[t]/\partial t$ at the $t^{th}$ time step. Notably, $x_j[t]$ is mathematical equivalent to the nonlinear model (Eqs. (10) and (11)) used by Bohm *et. al.*^21^ except for the noise term. To exploit the nonlinear dynamics, we simulate uncoupled spins for 50 time steps and the results are shown in Fig. 2a–d. Figure 2a,b show the representative trajectories under different energy landscapes with different feedback gains. When the feedback gain is below the threshold, the landscape of the Hamiltonian has only one energy minimum, and the Ising spins are squeezed within the vacuum state. As the feedback gain increases above the threshold, the landscape of the Hamiltonian will have two energy minima. Eventually, the Ising spins will bifurcate into two coherent states, corresponding to spin-up and spin-down. The real part of the reduced dynamics of 50 Ising spins are shown in Fig. 2c, where $x_{j,1}=0$ corresponds the vacuum state and $x_{j,2}=-x_{j,3}$ correspond to the coherent states. The implication is described in Fig. 2d. When the feedback gain is below their threshold, the stable fixed points are at $x_{j,1}=0$. When the feedback gain is above the threshold, the fixed points at $x_{j,1}=0$ become unstable whereas symmetric stable fixed points appear at $x_{j,2}=x_{j,3}$. Furthermore, from Eq. (4), we observe that the imaginary part of the reduced dynamics remains around 0 under perturbation of 0-mean Gaussian white noise. Figure 2e–h also show the energy landscapes and representative trajectories of the real part of the dynamics of uncoupled/coupled Ising spins. Similarly, when the gains are below/above the threshold, the Ising spins enter the vacuum/coherent states depending on whether or not they are coupled.

The evolution and expansion steps (see “Methods” section) in our CITS algorithm are based on two primal heuristics, CIM and SA. The intuition of cooperating these two algorithms is from the symmetric energy landscapes and trajectories shown in Fig. 2c, d. An observation is that when the spin $x_j$ is flipped to $-x_j$, the reduced dynamics ${\partial (-x_j)}/{\partial {}t}$ will instead be $-{\partial {}x_j}/{\partial {}t}$ (Eq. (3) are odd functions). Hence, the spin $x_j$ will go to the other stable fixed point. The blue dash line reveals that flipping the sign of the Ising spin does not affect the bistability of the reduced dynamics. During the evolution and expansion step, CITS can explore along the real coordinate space based on Eq. (4) while expanding the search space along the Stone space based on Eq. (7). As a result, CITS can boost CIM toward exploring multiple search spaces in the future time steps without losing the bistability. Figure 1c and dash lines in Fig. 1e shows the coherent Ising tree with depth $d=2$ and breadth $b=2$ at time step *t*. Compared to four identical and independent trajectories in Fig. 2f, the search space coverage of a coherent Ising tree is even broader, which gives an intuition that CITS can help escape out of local minima.

### Ablation study: complete and naive search schemes

**Figure 3:**
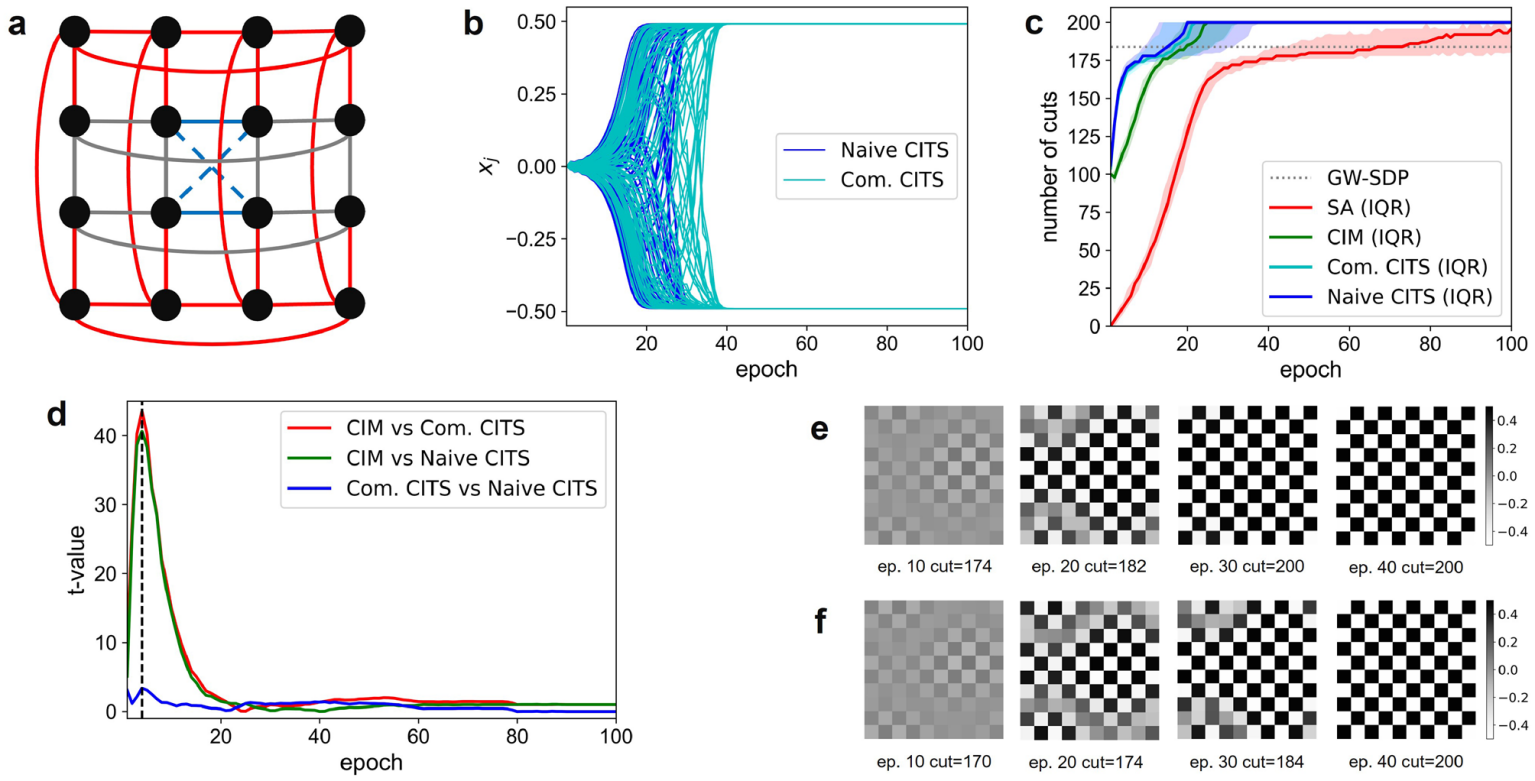
Benchmark results on square lattice graphs: **(a)** Graph structure of grid lattice with two periodic dimensions (all solid lines). Circular ladder can be obtained by only choosing the vertices connected by the edges shown in blue and gray solid line. And Mobius ladder can be obtained by twisting the blue solid lines to blue dash lines in circular ladder graphs. **(b)** A simulation of time evolution of spin amplitude on a 10-by-10 square lattice using two schemes (with feedback strength $\alpha =0.25$, and coupling strength $\beta =0.29$). **(c)** The number of cuts on 10-by-10 square lattice given by SA, CIM, CITS for 100 epochs. **(d)** The t-value between distributions of the number of cuts given by CIM, CITS for 100 epochs. **(e)** The Ising spin configuration at epoch 10, 20, 30 and 40, where two end of the color bar represent two stable solution of the oscillator (black for spin-up and white for spin-down) for naive scheme and **(f)** complete scheme.

To evaluate the efficiency of CITS as compared to SA and CIM, we run the simulations for 100 epochs for each instance. First, we test on a 10-by-10 square lattice with periodic dimensions. In the “Methods” section, we proposed two expansion (and exploration) schemes for CITS. The naive scheme only considers the search spaces (tree nodes) generated by the primal heuristic, SA. The complete scheme explores around the tree nodes based on the primal heuristic, CIM. In Fig. 3b, we show the time evolution of amplitudes corresponding to the 10-by-10 square lattice graph (Fig. 3a) of the two exploration schemes. Our simulations show that the initialization for CIM and CITS affect the evolution of the dynamics in subsequent epochs. Thus, we set the initialized points of the two methods to be the same in each run for a fair comparison. The impact of the initialization is beyond the scope of the proposed tree search algorithms and is left out of this article (In this work, we consistently assume the distribution of initialization subject to the noise applied to the CIM). After initialization around the unstable fixed point $x_1=0$, CITS explores the search space and eventually stabilizes at two symmetric fixed points $x_2=-x_3$ representing spin-up and spin-down. Figure 3e,f shows the explicit spin configurations at epoch 10, 20, 30, 40 corresponding to the time evolution of the spin amplitudes. In the first few epochs, the spin configurations form an organized region at the middle right of the square lattice. After around 10 epochs, the amplitudes of Ising spins bifurcate, which gives rise to the coupling effect. In the naive scheme, the spin configuration at epoch 20 is sub-optimal. At epoch 30, the system stabilizes at the global energy minimum for the rest of the time step. In the complete scheme, the convergence speed is slower but it eventually reaches the ground state at epoch 40.

Although a single simulation result is insufficient for claiming one scheme is better than the other, the visualization of the underlying dynamics may be able to provide some insights into the optimization process. 100 simulations were run for both schemes to evaluate their convergence speeds. Figure 3c shows the number of cuts on the 10-by-10 square lattice given by the solution at each epoch. To further evaluate the performance of CITS, we benchmark this result with SDP, SA and simulated CIM with the setup mentioned in the “Methods” section. Since we are comparing the performance between annealing-based heuristic algorithms, the approximate solution of 184 given by SDP is used as a baseline to evaluate the epochs-to-solution and the success rate of the proposed CITS algorithm and its two primal heuristics, SA and simulated CIM. Each of these three algorithms was run 100 times with different random seed numbers. Since the performance of each run varies, we studied the interquartile range (IQR, range between the 25th percentile $Q_{25}$ and the 75th percentile $Q_{75}$) in Fig. 3c, where the solid lines represent the 50th percentile $Q_{50}$. The epochs-to-solution are also shown in Table 2 to allow us to quantify the performance of each method.

**Table 2:** The epochs-to-solution for $10 \times 10$ square lattice graph when using SA, CIM and CITS of the 25th, 50th, 75th percentile. Both global optimum (to exact solution) and near-optimal local optimum (to approx. solution) are evaluated.

Methods	SA			CIM			CITS (complete)			CITS (naive)		
Percentile	$Q_{25}$	$Q_{50}$	$Q_{75}$	$Q_{25}$	$Q_{50}$	$Q_{75}$	$Q_{25}$	$Q_{50}$	$Q_{75}$	$Q_{25}$	$Q_{50}$	$Q_{75}$
To exact sol.	79	>100	>100	19	25	33	14	22	32	13	20	38
To approx. sol.	41	67	>100	15	20	26	10	17	26	9	15	32

In the beginning, CITS and CIM are initialized at a vacuum state, and suddenly obtain a 0.5-approximation solution due to the diffusion term introduced by the Gaussian white noise. Notably, during the first 7 epochs, the increment in the number of cuts for CITS and CIM are around 10 and 5 per epoch, respectively. CITS has a faster convergence to an approximate solution because deeper nodes in the coherent Ising tree tend to explore the tree in the coming future time step and based on the energy change, CITS decides the best move among the search space. At the beginning of the annealing process, the spin configurations are chaotic and a random flip tends to lower the Ising energy. Consequently, CITS tends to accept the further time steps. For the complete scheme, a fine-grained local search on each node is performed. As CITS goes from the root node to one of the leaf nodes, its primal heuristic CIM needs to be run for *d* times within an epoch. Since the tree depth is 2, CITS achieves approximately 2$\times$ faster convergence to the lower energy Hamiltonian as compared to CIM. Notably, the naive scheme only performs a coarse-grained local search on each node, where the primal heuristic CIM is not run to explore the surrounding search space. Intuitively, the fine-grained local search on each node provides more information of the Ising system, *i.e.* the reduced dynamics.

To evaluate the solution quality, we study the *t*-value ($t_{a,b} = {|\mu _a-\mu _b|}/{\sqrt{{\sigma _a^2}/{n_a}+{\sigma _b}/{n_b}}}$) between CIM and two schemes of CITS in Fig. 3d. $\mu _a$, $\mu _b$ are the mean values, $\sigma _a^2$, $\sigma _b^2$ are the variations and $n_a$, $n_b$ are the sizes of set *a* and set *b*. Observe that, at the first 4 epochs, the *t*-value between CITS and CIM increases significantly, which is consistent with the observation of “faster convergence”. We also observe that the *t*-value between two schemes in CITS is significantly smaller than the *t*-value between CITS and CIM. This indicates that the quality of the solutions generated by the naive and complete schemes in CITS are not statistically different. Thereafter, we may deduce that instead of fine-grained local search spaces, the breadth and the depth of the tree are the key contributors to the performance of CITS. Since the naive scheme saves a lot of computational complexity, the simulation of CITS is based on the naive scheme in the rest of this paper.

After a few epochs, the landscape of the Ising Hamiltonian becomes more complicated. In this scenario, both algorithms tend to be trapped in the local minima and slow down the annealing process, which takes 9/15/32 epochs (for $Q_{25}$/$Q_{50}$/$Q_{75}$) for CITS to outperform SDP and 13/20/38 epochs to reach the global optimum. For comparison, CIM needs 15/20/26 epochs to outperform SDP and 19/25/33 epochs to reach the global optimum, respectively. From Fig. 3d, we can see that 24 epochs are needed before CIM can generate solutions of comparable quality to CITS.

The speed of convergence of CITS depends not only on the “depth” of the coherent Ising tree but also on the “breadth”. A wider coherent Ising tree provides a larger search space, which potentially contains a spin configuration with a lower energy Hamiltonian. As mentioned in the “Introduction” section, the expansion of the coherent Ising tree is based on another primal heuristic SA, which is able to identify the most promising search spaces based on the highest flipping probability computed using Eq. (7). Hence, we also compared CITS with SA, where 79 epochs are needed to reach the ground state for $Q_{25}$. Note that the algorithms do not always outperform SDP within 100 epochs, where it takes 41/67 epochs for $Q_{25}$/$Q_{50}$. The Ising spins of SA do not have the properties of the “vacuum state” since SA initializes the spin configuration as all spin-up (or all spin-down) with 0 cut in the beginning. The number of spins allowed to be flipped is limited so as to preserve the quasi-equilibrium distribution given by the approximation and ensure that the Markov process will converge to a stable distribution. Nonetheless, it can outperform SDP and may possibly return a near-optimal solution in a longer but acceptable time scale.

### Benchmarking CITS on different MAX-CUT instances

In this section, we test SA, CIM and CITS on MAX-CUT instances using square lattice, circular ladder and Mobius ladder graphs. The parameters used in the simulation are shown in Table 3:

**Table 3:** The parameters used in the simulations.

Methods	Temperature(T*)	Feedback gain($\alpha$)	Coupling gain($\beta$)	Initialization	Noise
SA	Eq. (9)	/	/	All spins up	/
CIM	/	0.25 (lattice)0.07 (ladder)	0.29 (lattice) 0.39 (ladder)	$\mathbb {N}(0,10^{-2})$	$\mathbb {N}(0,10^{-2})$
CITS	Eq. (9)			$\mathbb {N}(0,10^{-2})$	0

**Figure 4:**
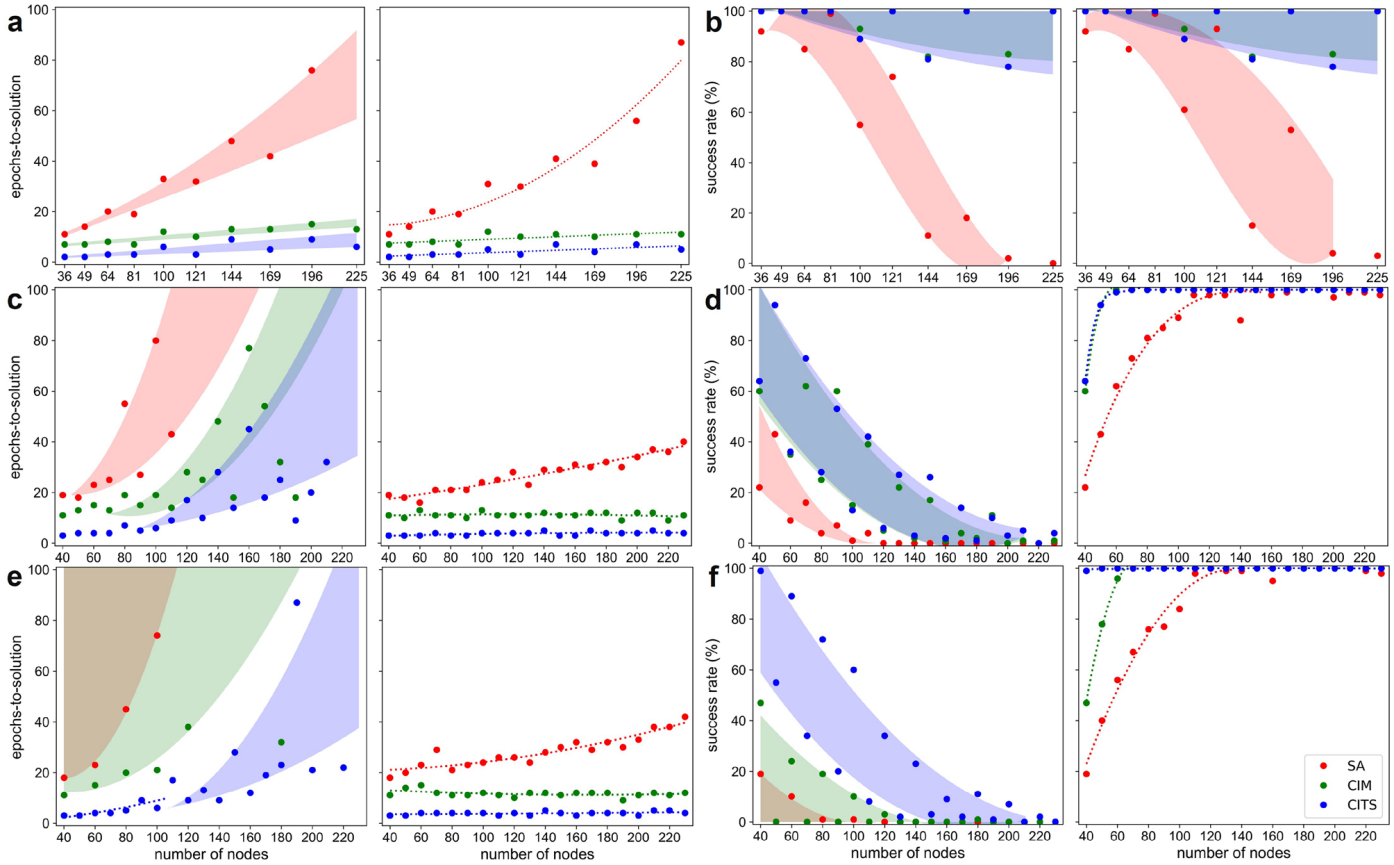
Benchmark results on square lattice graphs, circular ladder graphs and Mobius ladder graphs: **(a)** Epochs-to-solution of the the best reported result, and **(b)** success rate on square lattice graphs with different graph size. The targets of left sub figures are the exact solutions and of right figure are the approximate solutions given by SDP. (parameter used: $\alpha =0.25, \beta =0.29$). **(c, d)** are evaluate on circular ladder graphs, and **(e, f)** are evaluate on Mobius ladder graphs ($\alpha =0.07, \beta =0.39$).

Due to their parallelism, CITS and CIM simulation demonstrate strong potential in solving 10-by-10 square lattice graphs (Fig. 3a), which are regarded as easy instances since the Ising spins do not compete with each other. As a result, the graphs have only two naive solutions (alternative arranged, $S=(s_1,s_2,...,s_n)$ and $(-s_1,-s_2,...-s_n)$) that are regular graphs. However, if the side length of the square lattices is odd, the adjacent Ising spins may compete with each other leading to disorder patterns different from Fig. 3b. These graphs are known as frustrated graphs in which their Hamiltonians usually have more than two ground states. Square lattice graphs with side lengths ranging from 6 to 15 are studied in this section. The left figure in Fig. 4a shows the epochs-to-solution toward the global minima of the best reported result, where they indicate how fast the heuristic algorithms can escape the local energy minima. We observe that regular graphs usually require more epochs to find the ground state (upper bounds of the filled area) than frustrated graphs (lower bounds of the filled area). The epochs-to-solution of SA scales as a polynomial function of the number of nodes and the fitting curve corresponding to the regular graphs has a larger higher order coefficient because they only have two global energy minima whereas the frustrated graphs have multiple energy minima. For CIM and CITS, the epochs-to-solution scales linearly with the number of nodes, and the difference between fitting curves of regular graphs and frustrated graphs is insignificant. They also benefit from the continuous number representation of Ising spins. SA suffers from polynomial scaling due to the discretized representation of the Ising spin, which limits the number of spin flips per epoch. To gain more intuition on the slower convergence when using SA as compared to the two other algorithms, let us consider a derandomized and serialized version of SA: the Hopfield neural network (HNN)^27^ , which maximally switches one spin at one time. In the best-case scenario, it will require $2n^2$ time flips to solve for a $2n\times 2n$ square lattice. Notably, CITS usually has a lower zero-order coefficient in the fitting curves since a deeper coherent Ising tree tends to make decisions based on future time steps. However, the depth and breadth are only 2 in this case and the benefit of shorter ground-state search time diminishes as the number of nodes is increased. Thus far, our results show that CITS has 2.52×/6.38× speedup compared to CIM/SA to find the ground states of the square lattice graphs. The left figure in Fig. 4b shows the success rates of each heuristic algorithm within 100 epochs, revealing how likely that the annealing algorithms get trapped in the local energy minima and return sub-optimal solutions. The success rates of CIM and CITS are similar, achieving almost 100% on the frustrated graphs and decreasing logarithmically on the regular graphs while remaining above 80%. Theoretically, SA can be expected to always find the ground state if given an infinite time and hence, its success rate is closely related to the epochs-to-solution. SA can achieve a 100% success rate on square lattice graphs with side lengths 7 or 9. However, the success rate decreases polynomially on the frustrated graph. Similar to the epochs-to-solution, the success rates on regular graphs are also lower than the frustrated graphs.

In many cases, a near-optimal solution is acceptable when the time to find the exact solution is incredibly large. Thus, we also benchmarked these three annealing algorithms to the approximate solutions given by SDP, as shown in the right figures in Fig. 4a,b. SDP is able to achieve the exact solution for square lattice with the side length of lower than 10, and can only achieve an approximate solution for larger graphs. For CIM and CITS, the probability of finding near-optimal solutions are similar to those for finding exact solutions of the square lattice, which reveal that they are unlikely to be trapped in local minima in square lattice graphs. In this case, CITS achieves 2.55x speedup over CIM to find the approximate solutions. Meanwhile, SA shows a faster convergence speed to find approximate solutions compared to the exact solutions (which is obvious), especially for the regular graphs. However, it is still 6.38x slower than CITS. Since the performance of SA is limited by the speed, relaxed targets are more easily obtained at fewer epochs-to-solution.

In this section, we also benchmark CITS on other representative MAX-CUT instances (such as circular and Mobius ladder graphs) with the number of nodes ranging from 40 to 230, where some of them are regular graphs and some are frustrated graphs. The graph structure of the circular ladder consists of two concentric *n*-cycles and each pair of nodes are connected to each other and the adjacent nodes. Figure 3a shows that it can be considered as a special case of the rectangular lattice with one dimension only having a side length of 2 and the other dimension is periodic. On the left of Fig. 4d, it is clear that among the circular ladder graphs with a different number of nodes, CIM and CITS can only achieve a success rate above 75% on 60 node instances. Moreover, as the number of nodes increases, the success probabilities will further decrease. However, CITS still has 0.15%/11.50% improvement to finding exact solutions compared to CIM/SA. So we adopt the first percentile instead of the twenty-fifth percentile results for evaluating the speed of the algorithms. The results are shown on the left of Fig. 4c. Compared to the results in Fig. 4a,b, circular ladders can be considered a more difficult graph topology for MAX-CUT problems, where the fitting curve of epochs-to-solution and success rates of CIM and CITS both shows exponential scaling. On average, CITS can achieve 1.97$\times$/2.71$\times$ speedup of finding exact solutions on circular ladder graphs in terms of epoch-to-solution.

The approximate solutions given by SDP are far from the exact solutions, especially when the graph size is large. Therefore, these targets become easier to achieve for the annealing algorithms. Moreover, SDP performs worse in difficult instances. On the right of Fig. 4c,d, the curves corresponding to regular graphs and frustrated graphs are fitted jointly. Interestingly, in Fig. 4c, the epochs-to-solution of CIM and CITS are nearly constant whereas SA shows linear dependence on the number of nodes. The right figure in Fig. 4d shows that the success rates are better because SDP performs worse on a larger size graph and gives a relaxed target. When the number of nodes is above 60, CIM and CITS can achieve around 100% success rate of finding approximate solutions whereas SA has a lower success rate and is limited by the speed. On average, CITS can achieve 3.02$\times$/7.21$\times$ speedup as compared to CIM/SA and 2.40%/19.90% improvement in success rate to finding approximate solutions.

By twisting the blue dash lines in the circular ladder in Fig. 3a, the graph becomes a Mobius ladder, which is a cubic graph with all the adjacent and opposite nodes connected. If the number of vertices divided by 2 is odd, the Hamiltonian of cutting the Mobius ladder is minimized when the Ising spins are in an alternate arrangement of up and down along the ring. When the number of vertices divided by 2 is even instead, all the Ising spins are competing with the corresponding opposite spin in alternate arrangement spin configuration. The result in Fig. 4e,f reveals that the Mobius ladder graphs are harder to solve than the previously mentioned graphs. However, on average, CITS can achieve 3.12$\times$/7.45$\times$ speedup as compared to CIM/SA to find approximate solutions and 3.42$\times$/8.27$\times$ to find exact solutions on the Mobius ladder graph. For the success rate, CITS has 3.90%/14.60% improvement in finding approximate solutions and 21.35%/25.00% improvement in finding exact solutions.

## Discussion

We have proposed a heuristic search algorithm for Ising formulated combinatorial optimization problem, which we call CITS. This algorithm is inspired by the high-level idea of MCTS, which obtains a solution by exploring and exploiting the search space in the feasible regions. However, for NP-optimization problems, the size of the feasible regions scales exponentially with the size of the problem and therefore, it becomes infeasible to exhaustively explore and exploit the search space in the feasible regions. To address this issue, our (recursive-)depth limited search scheme aims to find the most promising feasible search spaces determined by primal heuristic SA. Note that our algorithm is an extension of the poor man’s CIM, which is a quantum-inspired algorithm with the measurement-feedback scheme. We combined the two primal heuristics by mathematically formulating the bistability of parametric oscillators in the Lagrange picture. In our implementation, we proposed two search schemes. The first scheme explores (using the primal heuristic CIM) every feasible region while expanding the Ising tree. The other scheme is a naive scheme that is the same as the first scheme except that the exploration step is removed. This reduces the computational complexity and was shown to be more computationally efficient in the section discussing the ablation study. Our results reveal that the advantage of CITS is due mainly to the breadth and the depth of the tree instead of exploring every local search space. To benchmark the performance of CITS as a general solver on Ising formulated problems, we evaluate its performance on MAX-CUT problems, where the instances have nodes ranging from 36 to 230. CITS has improvement in both epochs-to-solution and success rate as compared to the simulated poor man’s CIM on both square lattice graphs, circular ladder graphs, and Mobius ladder graphs, where CITS has maximally up to 3.42$\times$ speed up and 21.35% higher success rate.

We are aware that the quantum noise is related to the bifurcation^14^. It is not specified in the poor man’s CIM^21^, so we applied Gaussian white noise in our simulation. The implementation of CITS that interfaces with physical CIM is left out of this work. However, note that when physically implementing CIM with the measurement-feedback scheme, the delay of each round trip is mainly dominated by the communication between FPGA and the optical system. Thus, it is reasonable to expect that the speed up in epochs-to-solution is similar to the speed up in computation time.

We may expect to obtain a certain speed up by extending CITS to other combinatorial optimization problems like knapsack problems and travelling salesman problems, which are more practical for real-world problems. These Ising formulations of these problems have been demonstrated^4^. Even though CIM and CITS still expect to obtain sub-optimal solutions in polynomial time, the complexity will be higher. Thereby, it would not be trivial to implement on CIM and CITS due to the fact that problems like knapsack problems or travelling salesman problems contain equality constraints or inequality constraints. The demonstration of more real-world practical problems will be left for future work.

## Methods

### Problem mapping and baseline

MAX-CUT problems belong to the NP-hard class^28^, which means any NP problem can be mapped to the MAX-CUT problem in polynomial time. The description of the MAX-CUT problem is as follows. Given an undirected graph $G=\left( V,E\right)$, partition *G* into two complementary graphs, $G_1$ and $G_2$, such that the number of edges between $G_1$ and $G_2$ is maximized. The objective function can be written as:

5 $$\begin{aligned} \text {CUT}\left( s_1,s_2,\ ...,s_n\right) =\sum _{\left( i,j\right) \in E}\frac{1-s_is_j}{2} \end{aligned}$$

Comparing Eqs. (1) and (5), we observe that maximizing $\text {CUT}\left( s_1,s_2,\ ...,s_n\right)$ is equivalent to minimizing $H\left( s_1,s_2,\ ...,s_n\right)$, when $J_{ij}=1,\ \forall \left( i,j\right) \in E$.

For most MAX-CUT problems, there is no guarantee that exact solutions can be found in polynomial time. Generally, an acceptable solution (one that outperforms some baseline) found within an acceptable time (in polynomial time) is sufficient. In this work, Goemans-Williamson Semidefinite programming (GW-SDP), a 0.879-approximation algorithm for the MAX-CUT problems^29^, is chosen as the baseline algorithm for generating the targets with which to evaluate the efficiency of CITS or its primal heuristics. SDP is known as an approximation algorithm that relaxes integer linear programming problems in Eq. (5) to:

6 $$\begin{aligned} \text {CUT}\left( \sigma _{11},\sigma _{12},\ ...,\sigma _{nn}\right) =\sum _{\left( i,j\right) \in E}\frac{1-\sigma _{ij}}{2} \end{aligned}$$

$\sigma _{ij}=s_is_j\in \left\{ -1,1\right\}$, represents whether two vertices are in the same subgraph or not. The constraints of SDP are: $\sigma _{ii} = 1$, $\sigma _{ij} = \sigma _{ji}$, and $\Sigma = \left[ \sigma _{ij}\right]$ is positive semidefinite. As a result, the MAX-CUT problems are transformed into a special case of convex programming, in which the *cvx* solver^30^ can efficiently maximize the objective function in Eq. (6) with the aforementioned constraints. After transformation, a Cholesky factorization may be performed on $\Sigma =S^{T}S$. A projection of $S\approx \text {sgn}\left[ \Sigma B\right]$ via random rounding may then be utilized to approximate the solution. *B* is an $n\times {}m$ random matrix, where *m* columns represent *m* random planes to be projected on to generate approximate solutions. Finally, among all approximate solutions, the one giving the largest number of cuts will be selected.

### Primal heuristic 1: Parallel SA

The stochastic spin update scheme in SA is inspired by thermal annealing, where the probability of the spin-flip is determined by quasi-equilibrium distribution based on the Metropolis-Hasting^31^ algorithm:

7 $$\begin{aligned} p_i=\frac{1}{Z}\exp \left( -\frac{\Delta H_i}{k_BT}\right) \end{aligned}$$

$p_i$ is the flipping probability of the *i*-th spin, *Z* is a normalization factor, $k_B$ is the Boltzmann constant, *T* is the annealing temperature parameter, and $\Delta H_i$ is the energy difference due to flipping the *i*-th spin. Note that the ergodic search of all possible spin configurations requires a $1\times 2^n$ probability transition matrix. As a result, following the exact Boltzmann distribution becomes difficult. Instead, a quasi-equilibrium distribution only concerning the flipping probability of each spin is utilized. In this scenario, the normalization term is approximated as $Z = \sum _{i=1}^n \exp \left( -\frac{\Delta H_i}{k_BT}\right)$. Typically, the energy change in flipping the $i^{th}$ spin is:

8 $$\begin{aligned} \Delta H_i = -2s_i\sum _i^nJ_{ij}s_j \end{aligned}$$

To speed up the algorithm, we adopt a synchronous update by approximating Eq. (7) to $p_i \leftarrow T^*p_i/\sum _{i}^{n}p_i$. Note that the quasi-temperature parameter, $T^*$, is initialized as 1 and subject to a temperature scheduling scheme^32^ as follows. Increase the temperature significantly in the first few epochs to provide thermal energy to escape the minimum. Thereafter, the temperature is aggressively decreased in the next few epochs before slowing the temperature decrease to gradual decay.

9 $$\begin{aligned} \Delta T^*(t+1) = {\left\{ \begin{array}{ll} 1.05T^*(t), &{} t\le \frac{N_{epochs}}{4}\\ 0.95T^*(t), &{} \frac{N_{epochs}}{4}<t\le \frac{N_{epochs}}{2}\\ 0.99T^*(t), &{} t>\frac{N_{epochs}}{2}\\ \end{array}\right. } \end{aligned}$$

### Primal heuristic 2: Simulated Poor man’s CIM

Another primal heuristic is CIM, which encodes the coherent Ising spin state as the phases of degenerate oscillators. With mutual injections of the signals between each oscillator, the CIM will oscillate in one of the approximate ground states, thereby giving near-optimal solutions to the combinatorial optimization problem. The mutual injections are usually realized by the network of delay lines^19^, or approximated by measurement-feedback scheme^18^ where the latter is discretized by the Euler method. The simulation in the present work follows the poor man’s CIM^21^, where the time evolution of the *i*-th spin at time step *t* is:

10 $$\begin{aligned} x_i\left[ t+1\right] = \cos ^2\left( f_i\left[ t\right] -\frac{\pi }{4}+\xi _i\left[ t\right] \right) - \frac{1}{2} \end{aligned}$$

$x_i\left[ t\right]$ is the measurement of each Ising spin at time step *t*, and $\xi _i\left[ t\right]$ is the diffusion introduced by modelled using Gaussian white noise. Note that the noise for poor man’s CIM is inside the trigonometric function whereas it is outside for CITS. For CIM, the noise is modelled as $\xi \sim \mathbb {N}(0,10^{-2})$, and the same level of noise is applied at the beginning for random initialization and removed at the later time steps for deterministic convergence. $f_i\left[ t\right]$ is the feedback term injected back to each of the Ising spins:

11 $$\begin{aligned} f_i\left[ t\right] = \alpha x_i\left[ t\right] +\beta \sum _{j}^{n}{J_{ij}x_j\left[ t\right] } \end{aligned}$$

The feedback gain, $\alpha$, and coupling gain, $\beta$, remain the same for both CIM and CITS, but should be chosen carefully to ensure bifurcations of Ising spins. After trial-and-error, we select $\alpha /\beta$ for the 2D square lattices as 0.25/0.29, and as 0.07/0.39 for circular ladders, Mobius ladders.

### High-level strategy: CITS Algorithm

**Figure 5:**
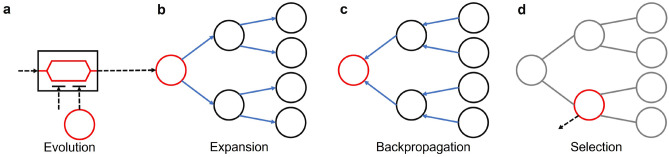
An illustrative workflow of CITS with tree depth of $d=2$ and breadth of $b=2$: **(a)** Ising spin evolution in simulated (or physical) CIM, the reduced dynamics of the oscillators tends to approach lower Hamiltonian. **(b)** Expansion of coherent Ising tree is based on the flipping probability given by the primal heuristic SA, where the most potential flipping will be expanded as a child node. **(c)** CITS computes the rewards *R* of each node based on the Ising Hamiltonian of the corresponding spin configuration. And the return *Q* of the child nodes will backpropagate to the parent nodes for computing their return. **(d)** CITS select the nodes with highest return successively, until reaching the leaf node, or all the child nodes have negative returns.

The flow of CITS (Fig. 5) consists of four main steps.

1. Evolution: As shown in Fig. 5a, the Ising formulated graph is encoded onto the simulated CIM (or its physical implementation). The parametric oscillators are then allowed to interact with each other while evolving. The obtained (measured) result from the CIM is then used to initialize the root node of the coherent Ising tree.
2. Expansion (and Exploration): For all nodes in the current layer, compute the switching probability, $p_i$, according Eq. (7) (the primal heuristic SA). Thereafter, create *b* child nodes based on top-*b* probabilities, which will be further given to the created child nodes as the priors. Note that the *i*-th spin in the corresponding child node will take the value of the opposite number, *i.e.*, $X_i^{(k)} = \left( x_1, x_2, ..., -x_i, ..., x_n\right)$. Afterward, the child nodes will explore the search space using the primal heuristic CIM (Eq. (3)). A naive version to reduce the computational complexity is obtained by removing the exploration step. The expected reward of each node in both schemes may be computed by:
  12 $$\begin{aligned} R_i^{(k)}=H\left( \text {sgn}[X_i^{(k)}]\right) -H\left( \text {sgn}[X^{(0)}]\right) \end{aligned}$$
  where $X_i^{(k)}$ is the spin configuration corresponding to the *i*-th node in the *d*-th layer. This step is repeated for these child nodes if they have not reached the tree depth *d*;
3. Backpropagation: Starting from the nodes at the deepest layer, CITS samples the return, $Q_j^{(k)}$, of each node. The return is successively backpropagated to their parent nodes (following the blue arrow lines in Fig. 5c) until the root node is reached. The return of the *j*-th node in *k*-th layer is computed based on the prior $p_j^{(k)}$ and the expected reward $R_j^{(k)}$, as well as the prior $p_i^{(k+1)}$ and the return $Q_i^{(k+1)}$ of the child nodes as:
  13 $$\begin{aligned} Q_j^{(k)}=p_j^{(k)}R_j^{(k)}+\sum _{i\in C_j^{(k)}}{p_i^{(k+1)}Q_i^{(k+1)}} \end{aligned}$$
  where $C_j^{(k)}$ is the set containing all child nodes of the *j*-th node in *k*-th layer;
4. Selection: Starting from the root node to a chosen layer, select the child node with the the highest return $Q_j^{(k)}$ successively. If the current node does not have any child node with a positive return, stop selection. The spin configuration of the selected child node is given to the simulated (or physical) CIM for the evolution in the next time step.

## Data Availability

The data that support the results of this study are available from the corresponding author upon reasonable request.
